# ACE2 Is an Adjacent Element of Atherosclerosis and COVID-19 Pathogenesis

**DOI:** 10.3390/ijms22094691

**Published:** 2021-04-29

**Authors:** Anastasia V. Poznyak, Evgeny E. Bezsonov, Ali H. Eid, Tatyana V. Popkova, Ludmila V. Nedosugova, Antonina V. Starodubova, Alexander N. Orekhov

**Affiliations:** 1 Institute for Atherosclerosis Research, Skolkovo Innovative Center, 121609 Moscow, Russia; 2 Laboratory of Cellular and Molecular Pathology of Cardiovascular System, Institute of Human Morphology, 3 Tsyurupa Street, 117418 Moscow, Russia; evgeny.bezsonov@gmail.com; 3 Laboratory of Angiopathology, Institute of General Pathology and Pathophysiology, 8 Baltiiskaya Street, 125315 Moscow, Russia; 4 Department of Basic Medical Sciences, College of Medicine, QU Health, Qatar University, Doha P.O. Box 2713, Qatar; ali.eid@qu.edu.qa; 5 Biomedical and Pharmaceutical Research Unit, QU Health, Qatar University, Doha P.O. Box 2713, Qatar; 6 V.A. Nasonova Institute of Rheumatology, 34A Kashirskoye Shosse, 115522 Moscow, Russia; popkovatv@mail.ru; 7 Federal State Autonomous Educational Institution of Higher Education I.M. Sechenov First Moscow State Medical University (Sechenov University), Ministry of Health of the Russian Federation 8/2 Trubenskaya Street, 119991 Moscow, Russia; profmila@mail.ru; 8 Federal Research Centre for Nutrition, Biotechnology and Food Safety, 2/14 Ustinsky Passage, 109240 Moscow, Russia; avs.ion@yandex.ru; 9 Pirogov Russian National Research Medical University, 1 Ostrovitianov Street, 117997 Moscow, Russia

**Keywords:** COVID-19, coronavirus disease, atherosclerosis, ACE2

## Abstract

COVID-19 is a highly contagious new infection caused by the single-stranded RNA Sars-CoV-2 virus. For the first time, this infection was recorded in December 2019 in the Chinese province of Wuhan. The virus presumably crossed the interspecies barrier and passed to humans from a bat. Initially, the disease was considered exclusively in the context of damage to the respiratory system, but it quickly became clear that the disease also entails serious consequences from various systems, including the cardiovascular system. Among these consequences are myocarditis, myocardial damage, subsequent heart failure, myocardial infarction, and Takotsubo syndrome. On the other hand, clinical data indicate that the presence of chronic diseases in a patient aggravates the course and outcome of coronavirus infection. In this context, the relationship between COVID-19 and atherosclerosis, a condition preceding cardiovascular disease and other disorders of the heart and blood vessels, is particularly interesting. The renin-angiotensin system is essential for the pathogenesis of both coronavirus disease and atherosclerosis. In particular, it has been shown that ACE2, an angiotensin-converting enzyme 2, plays a key role in Sars-CoV-2 infection due to its receptor activity. It is noteworthy that this enzyme is important for the normal functioning of the cardiovascular system. Disruptions in its production and functioning can lead to various disorders, including atherosclerosis.

## 1. Introduction

The coronavirus disease develops as a result of infection with a single-stranded RNA virus called Sars-CoV-2 that apparently passed to humans from a bat. This transition became possible as a result of a mutation of function enhancement in the spike (S) protein. This disease is primarily considered in the context of damage to the respiratory system, but complications affecting the cardiovascular system are also widespread. These complications include myocarditis, myocardial damage and subsequent heart failure, myocardial infarction, and Takotsubo syndrome [1,2].

The virus is highly transmissible from person to person, mainly in an airborne way. After invading the mucous membranes, the virus invades the lungs and causes a phase of viremia [3], entering the circulatory system. In this phase, symptoms such as cough and fever are most often noted. The high transmissivity of COVID-19 can be explained by the high viral load in the viremia phase, and an effective molecular mechanism that recognizes the binding protein angiotensin-converting enzyme 2 (ACE2), which allows infecting human alveolar epithelial cells [3,4].

During the acute phase, the immune system is involved in controlling the virus. Apparently, the first phase of viremia is followed by acute organ damage. This phase usually occurs 7 days after the first appearance of symptoms. At this point, the virus is ineffectively inhibited by the immune system. COVID-19 results in the suppress of the respiratory function through exploiting the ACE2 binding receptors in the acute phase, which leads to pneumonia. The burden of respiratory system impairments ranges from breath shortness and cough (mild) to acute respiratory distress syndrome (severe). The omnipresence of ACE2, as well as the vulnerability to COVID-19 are potentially linked to multiple organ failure, such as kidney and liver damage, which results in systemic disorders, and acute myocardial infarction, which leads to myocarditis. The immune system, triggered by virus replication, plays a crucial role in organ damage in the acute phase due to over-activation [5,6].

Among other events, an abnormal inflammatory response leads to an excess of cytokines and chemokines. The pathogenesis is associated with a high affinity of the virus to ACE2, while the alveolar epithelium, endothelium, macrophage, and lymphocytic cells are affected, which leads to apoptosis and pyroptosis in the immune cells [7].

## 2. Cardiovascular Health Determines COVID-19 Outcome

Increased troponin levels in COVID-19 are associated with more severe disease manifestation and mortality, contributing to the significance of myocardial damage as a prognostic factor.

Another independent prognostic factor of hospital death in patients suffering from COVID-19 is enhanced levels of N-terminal pro-brain-natriuretic peptide (NT-proBNP). This acts at lower levels than in heart failure [8], which suggests that relatively moderate changes in cardiac function may critically determine the outcome in COVID-19. In the Chinese cohort of COVID-19 patients, the incidence of arrhythmia was found to be surprisingly high [9]. No evidence of cardiomyocyte infection was observed, but the known SARS-CoV-2 receptor, angiotensin-converting enzyme 2 (ACE2), is expressed on the cardiomyocytes [10].

Moreover, the underlying cardiovascular disease can aggravate the development of COVID-19. The meta-analysis revealed the increased mortality among hospitalized COVID-19 patients with such comorbidities as cardiovascular disease (10.5%), diabetes (7.3%) or hypertension (6%) compared to those who had no underlying health issues. Such consistency was observed in the Chinese cohort, as well as patients from Italy and the US [11].

Increased D-dimer, which is a product of fibrin degradation levels, are a marker of adverse outcomes of COVID-19, and disseminated intravascular coagulation with a significant risk of venous thromboembolism (VTE) and ischemic stroke [12]. The cumulative frequency of VTE in 69% of cases, despite anticoagulant treatment, was reported by the ultrasound in 26 severe patients suffering from COVID-19 [13].

The frequency of ischemic stroke was higher in patients with a severe course of the disease compared to patients with milder COVID-19 symptoms. An association was found between six cases of ischemic stroke and systemic inflammatory changes, increased levels of D-dimer, and antiphospholipid antibodies that indicate hypercoagulation [14,15].

A hypothesis has been proposed by Bonow et al., which explains an increased susceptibility of patients with coronary heart disease (CHD) and risk factors for atherosclerotic cardiovascular disease to adverse outcomes and death from COVID-19 [16]. COVID-19 is an acute infection, and, similar to other diseases of this kind, hyperdynamic circulation caused by COVID-19 in patients with predisposing CHD factors can aggravate the unstable balance by elevating the myocardial oxygen demand, leading to acute coronary syndrome (ACS) [17]. According to the aforementioned hypothesis, an overproduction of cytokines may lead to the ACS resulting in instability and rupture of the atherosclerotic plaque [18].

Plasma concentrations of TNF-α, IL-6, and IL-1β are tightly linked to the progression and instability of atherosclerotic plaques. Moreover, the activation of proteases contributes to plaque rupture and lumen thrombosis due to a direct negative effect on the protective fibrous cap of the plaque [19]. An observational study, which was conducted to assess the long-term consequences of Sars-CoV infection, confirmed the effect of Sars-CoV on lipid metabolism and atherogenesis [20]. Twenty-five individuals, who recovered from Sars-CoV 12 years ago, were participating in this study. Results of the metabolomic analysis revealed the changes in lipid metabolism with a crucially elevated amount of free fatty acids, lysophosphatidylcholine, lysophosphatidylethanolamine, and phosphatidylglycerol. Notably, 11 participants also suffered from cardiovascular disease (CVD).

The pathogenesis of Sars-CoV disease and COVID-19 are very similar. Both diseases are distinguished by impaired immune system overactivation, resulting in cytokine overproduction [3]. The risk of restenosis in patients undergoing PCI with stent implantation due to coronary heart disease (CHD) can be increased in response to hyperactivation of proinflammatory patterns, generally characterized by a cytokine storm.

Apart from ACS, heart failure may also occur as a result of hemodynamic decompensation. Acute myocarditis can fulminate with no prior signs of CVD in a small proportion of cases, which is a result of the presence of ACE2 on cardiac myocytes. This may result in chronic dilated cardiomyopathy [18]. Shi et al. [21], as well as Guo et al. [2], described hospitalized patients who developed myocardial damage. Among signs of myocardial damage increased plasma levels of a highly sensitive troponin I (TnI) were described. Patients with elevated TnI are older and have a high prevalence of hypertension, diabetes mellitus, coronary arterial disease, and heart failure. More severe systemic inflammation has been described, including higher plasma white blood cell concentrations and CRP, resulting in a more complex respiratory pattern with a higher frequency of acute respiratory distress syndrome (ARDS) demanding assisted ventilation than in patients with no signs of myocardial damage. Thus, according to Shi et al. [21], in a total population of 416 hospitalized patients examined with a confirmed diagnosis of Sars-CoV, 82 patients exhibited signs of myocardial damage, which resulted in a mortality rate of 51.2% in this group, which is significantly higher than in patients without increased TnI (4.5%). Another study, conducted by Guo et al. [2], involved 187 patients with a confirmed diagnosis of Sars-CoV, among which patients with pre-existing CVD as a comorbid condition but without an increase in TnI revealed a worse outcome than patients without a comorbid condition, with a mortality rate of 13.30%.

Hemodynamic changes are the main contributor to the potential long-term effects of COVID-19, among which progression of atherosclerosis and, as a result, an enhanced risk of thrombosis. These may have an impact on the left ventricular systolic function and enhance retrograde pressure on the left ventricle, which leads to decompensation. The enhanced deep vein thrombosis (DVT) events rate, which is the result of an abnormal blood clotting, can result in more cases of pulmonary embolism and pulmonary hypertension. COVID-19 may directly affect the left ventricular systolic function and enhance retrograde pressure.

## 3. Atherosclerosis

Apart from COVID-19, cardiovascular diseases and pathologies are still the most widespread cause of death among the adult population around the globe [22]. Hypertension is considered a modifiable risk factor for cardiovascular diseases, and the reduction of blood pressure can decrease cardiovascular risk [22]. However, the reduction of blood pressure is the only approach, while cardiovascular diseases have a much more complex and still not completely clear origin [23].

Atherosclerosis is the main predictor for most cardiovascular diseases [24]. Two main components of atherogenesis are inflammation in response to injury of arterial wall and lipid metabolism alterations.

Endothelial dysfunction often underlies the development of atherosclerosis, along with the subsequent expression of cellular adhesion molecules (CAM), adhesion of circulating leukocytes to the endothelial cells [25], as well as migration of leukocytes and the fibrous cap formation around the lipidic core, which adversely impacts a vascular lumen [26]. The renin-angiotensin system (RAS) is also implicated in atherosclerotic lesion development and progression. This involves an impact on inflammation, balance of coagulation, fibrosis, functioning of endothelium, structural remodeling, and plaque stability [24,27].

The conventional RAS cascade, involving the renin-mediated conversion of angiotensinogen to angiotensin I (Ang-I), which is associated with the cleavage of Ang-I to angiotensin II (Ang-II) by the angiotensin-converting enzyme (ACE), is not the only one, which is important for atherogenesis. Other components of this system, other peptides, and enzymes are also implicated [27]. The most significant is suggested to be ACE2, which is normally liable for the production of angiotensin 1–7 (Ang-1–7) from Ang-II. The anti-inflammatory response, anti-thrombotic effects, vasodilation, and growth-inhibition are mediated via the opposition of the heptapeptide to Ang-II effects [27].

## 4. Atherosclerosis and COVID-19

Vascular endothelial cells are crucially involved in the maintaining of the hemostasis through regulating inflammation, vasomotor tone, and blood clotting factors [28]. COVVID-19 is spreading fast, and it becomes more and more obvious that vascular endothelium and the whole cardiovascular system are affected. The underlying mechanism of endothelial damage is the virus replication itself, which contributes to the diminished ACE2 regulation, exposing endothelial cells to Ang II in the absence of modulatory effects of angiotensin 1-7 (Ang 1-7) [29]. Data obtained from three COVID-19 patients demonstrated the viral incorporation into endothelial cells and accumulation of inflammatory cells [30].

The release of cytokines and chemokines is stimulated by endothelitis, which is an important part of the COVID-19 infection pathogenesis. This may result in coagulopathy accompanied by elevated fibrinogen, antithrombin, D-dimers, activated partial thromboplastin time (aPTT), increased prothrombin (PT), and other markers. In severe cases, disseminated intravascular coagulopathy (DIC) can also occur [31].

It is undoubtedly that the pre-existing cardiometabolic factors contribute to the severity of clinical manifestations of patients suffering from COVID-19. An intermediary condition is considered to be endothelial dysfunction. Thus, atherosclerosis, which shares risk factors such as cardiac troponin and diabetes mellitus, with severe COVID-19 infections, is a chronic inflammatory endothelial disease characterized by infiltration, deposition, and oxidation of lipids, activating and promoting a self-sustaining inflammation [32]. Early stages of atherogenesis are characterized by endothelial damage, which is accompanied by the accumulation of multiple-modified low-density lipoprotein (LDL) and other lipoproteins. This contributes to the inflammation of the arterial wall. Both the innate and adaptive immune systems play a crucial role in lesion formation and plaque characterization, supporting and contributing to the pro-atherogenic condition.

Cholesterol crystals and oxidized phospholipids obtain the properties of damage-associated molecular patterns (DAMPs). They can be recognized by Toll-like receptors (TLRs) and NOD-like receptors (NLRs), and this interaction triggers the NLRP3 inflammatory pathway, which leads to the proteolytic cleavage of pro-IL-1β and pro-IL18 to mature IL-1β and IL-18 [33]. Atherogenesis is accompanied by inflammation and cytokine overexpression, which are the consequences of the inflammatory signaling pathways TLR4/NF-κβ and JAK/STAT activation. All this contributes to innate and adaptive immune cell activation [34]. In such a way the pro-inflammatory state forms, which potentially increase the vulnerability to COVID-19.

IL-1β acts by increasing the local inflammation and promoting plaque instability through the induction of IL-6, TNF-α, IL-8, and other cytokines and chemokines. IL-1β, IL-6, and TNF-α, are also produced by non-classical atherosclerosis-activated CD14++ CD16+ monocytes, which are strongly correlated with disease progression. Similarly, the complement system is associated with atherosclerosis progression, being serum C3 and C4 levels, exacerbating inflammatory responses associated with an increased risk of CVD [35].

T-helper cells appeared to be the main CD4+ effectors in atherosclerosis, promoting the disease development via the stimulation of pro-inflammatory cytokines production [36]. What is more, B2 subsets of B cells are the main activated cells, which in turn enhance the adaptive immune response [32]. Interestingly, several studies [37,38] suggested an autoimmune response in atherosclerosis, which contributes to the transformation of regulatory T cells from the original protective phenotype (FoxP3+) to the pathogenic one (RORyt, T-bet, Bcl-6). Laboratory tests performed on samples from patients with a severe COVID-19 infection revealed elevated plasma levels of aforementioned cytokines and also levels of immune cells, similar to the atherosclerotic condition. Non-classical monocytes were suggested to play a key role [39]. This condition may contribute to the host’s susceptibility to cytokine storm development and worsening COVID-19 manifestations due to over-activation of the immunological response. Thus, Sars-CoV-2 infection is a potential stimulator of the already activated specific inflammatory pathways, such as inflammasome, JAK/STAT, and NF-κβ pathways, and alterations of the immune system are observed. This hypothesis can explain the severity of clinical course in patients with atherosclerosis, at least in part. It can be confirmed by the levels of tissue cholesterol that characterize atherosclerosis.

A relationship between the ability to adhere, protease activity or endocytosis, and the level of tissue cholesterol was demonstrated in the study performed on cell culture by Wang et al. [40]. They focused on different cells belonging to three groups: The young, the elderly, and the elderly suffering from chronic inflammation, showing increasing signs of age-related infectivity. Results of this investigation explain the higher incidence of the asymptomatic form of COVID-19 in children and, conversely, the higher replication of the virus in elderly patients suffering from chronic inflammation, which leads to negative outcomes. Chronic inflammation of the vessels is linked to the tissue macrophages overloaded with cholesterol, which increases the likelihood of acquiring a severe COVID-19 infection.

The role of chronic inflammation is considered crucial in atherogenesis due to the high levels of proinflammatory cytokines, such as IL-6, TNF-α, and IL-1β, and the progression and instability of atherosclerotic plaque. This makes them important targets for potential anti-atherosclerotic therapy [41]. Clinical studies focus on agents that inhibit the IL-1, IL-6, and TNF-α pathways to reduce the risk of coronary heart disease and reduce adverse outcomes after injury, which may also be potentially useful in the treatment of COVID-19 [32].

## 5. ACE2 Is an Adjacent Element

### 5.1. COVID-19

According to the pathogenesis of COVID-19, the role of ACE2 viral induction may be involved in reducing the production and shedding the receptor, leading to dysfunction of the renin-angiotensin system as well as increasing vascular permeability. Deficiency of ACE2, which is involved in the modulation of the renin-angiotensin-aldosterone system (RAAS), is associated with an increase in the level of angiotensin 2, which leads to well-known adverse effects, such as hypertension, exudation, hypertrophy, fibrosis, and, subsequently, to a more severe course of infection. These findings are supported by a documented ACE2 deficiency in populations of African descent, such as African Americans, which explains the high prevalence of severe symptoms and high mortality rates in such communities. The biological variability of ACE2 expression associated with adaptation to environmental conditions in black populations is associated with early hypertension, the progression of atherosclerosis, and cardiovascular disease, and can also manifest in two ways during COVID-19 [6].

Although ACE2 deficiency may limit adhesion to human cells and potentially act as a form of immunity against infection, once such patients got infected with COVID-19, they are more susceptible to the development of ARDS, sepsis, and multiple organ failure [39]. ACE2 participates in the inactivation of bradykinin des-Arg9, as a component of the kallikrein-kinin molecular system, causing angioedema in the lungs due to the effect of local vascular leakage [42]. In contrast, the role of anti-S-protein neutralizing antibodies in the relief of acute lung injury is controversial [6].

Laboratory results showed dysregulation in the immune system, manifested by a pro-inflammatory pattern affecting mainly the lung tissue, with the infiltration of a large number of cells. COVID-19 increases plasma secretion of interleukin 1β (IL-1β), interferon γ (IFN-γ), interferon-γ-induced protein 10 kDa (IP-10), monocytic chemoattractant protein-1 (MCP-1), IL-4, and IL-10. Activation of the complement system is detected in the pathophysiology of ARDS with an increase in the plasma level of C5a and directly during autopsy assessment, while the high presence of C3a and C3 fragments play a primary role and is potentially useful for effective therapy [43].

After a severe infection, the pathway of T-helper (Th) 1-lymphocytes is hyperactivated, causing excessive production of CD14++ and CD16+ inflammatory monocytes responsible for exacerbating inflammation and increasing the plasma concentration of IL-6 [10].

IL-6 is a key cytokine that triggers increased liver production of acute-phase proteins (APPs), such as C-reactive protein (CRP) and fibrinogen, causing hypercoagulable disease, which is characterized by thrombotic and embolic phenomena and can predict the severity of infection [44,45]. The clinical features of deep acute venous thrombosis and pulmonary embolism are probably associated with such disorders. IL-6 is a target for therapy with tocilizumab, a monoclonal antibody that has demonstrated efficacy in severe infections and is under trial for approval against Sars-CoV-2.

In addition to IL-6, high levels of IL-2, IL-7, IL-10, granulocyte colony-stimulating factor (GCSF), IP-10, MCP-1, macrophage inflammatory protein 1-alpha (MIP-1α), and TNF-α were recorded in the host plasma, representing a well-described cytokine storm. In ICU patients, this leads to a decrease in the plasma concentration of lymphocytes due to pulmonary aggregation and sequestration [6].

The pathogenetic mechanism that explains the various degrees of hyperactivation of the immune system, which can cause severe symptoms, is still doubtful. Nevertheless, analysis of statistics from Italy and China on severe infections or related deaths reveals some predisposing patterns, that may trigger higher susceptibility to COVID-19. Meta-analysis of data from 53,000 COVID-19 patients from Wuhan revealed the occurrence of 2% of severe disease manifestations and 3% of death. Risk factors for severity include the following: Male gender, age (over 60 years), and association with any comorbidities, including diabetes, hypertension, and cardiovascular disease [46].

### 5.2. Atherosclerosis

The first published data demonstrating the importance of ACE2 for atherosclerosis belongs to Zulli et al. [47]. In this study, the immunolocalization of ACE2 protein in macrophages and SMC actin-positive cells from rabbit atherosclerotic plaques was shown. After that, an anti-atherogenic role of ACE2 was confirmed in several experiments and observations [48,49].

The progression of early lesions in rabbits was found to be aggravated by overexpression of ACE2 on aortic plaques. In the study by Dong et al. [48], rabbits underwent endothelial injury and received an atherogenic diet. The potential mechanism of such an effect can be the transformation of Ang-II to Ang-1–7. This scenario includes a decreased local inflammation, as well as lowered rate of macrophage infiltration, MCP-1 expression, and lipid deposition, and also a plaque stabilization, caused by enhanced collagen content. Similar effects were observed in rabbits fed with a high-cholesterol diet. ACE2 acts in an anti-atherogenic way through the improvement of endothelial function and inhibition of proliferation and migration of vascular smooth muscle cells. Moreover, ACE2 upregulated PI3K-Akt pathway and downregulated Ang-ll/ROS/NF-κB signaling pathway, as well as JAK-STAT, ERK1/2, and p38 MAPK pathways [50]. Similarly, in ApoE-KO mice, ACE2 overexpression appeared to aggravate the size of atherosclerotic lesions, improve endothelial homeostasis, at least in part, via a mechanism involving an inhibition of Ang-II-induced production of reactive oxygen species [49]. Zhang et al. [51] identified the prevention of atherosclerotic plaque evolution due to the inflammatory response suppression. In ApoE-KO animals overexpressing ACE2, this can be expressed, for example, in the induction of cytokines and adhesion molecules expression, stimulated by Ang-II.

The use of the ACE2-deficient mice model (ACE2-KO) also supports the atheroprotective role of ACE2. In both ApoE-KO and LDL receptor-deficient mice (LDLR-KO) models, deficiency of ACE2 led to the bigger atherosclerotic lesions in comparison with the respective controls. Moreover, an enhanced susceptibility to atherosclerosis was found to be linked to the inflammatory profile within the plaque [52,53,54]. However, the atheroprotective role of ACE2 in humans is still not clarified.

The presence of ACE2 in human veins, arteries, both healthy and affected by atherosclerosis was identified by Sluimer et al. in 2008. Expression of ACE2 was also found in endothelial cells, smooth muscle cells (SMCs), and macrophages. Moreover, in both early and advanced human plaques, the mRNA and protein of ACE2 were observed. Due to the fact that the activity of ACE2 was detected to be lower in advanced lesions, even when the total protein expression of ACE2 appeared to be the same through all stages of the disease, allows the suggestion that the atherosclerosis development is accompanied by the differential regulation of ACE2 [55].

An enhancement in the initial activity of circulating ACE2 was identified in patients suffering from chronic kidney disease with atherosclerotic plaques in comparison to patients with no plaque by Anguiano et al. [56]. This finding suggests that the elevated activity of circulating ACE2 is linked to an enhanced risk for silent atherosclerosis. In accordance with this suggestion, elevated levels of circulating ACE2 protein were identified in women with CHD in comparison with the healthy group [57]. Such an effect was associated with multi-vessel lesions, which indicates the role of ACE2 in a compensatory mechanism in coronary atherosclerosis.

ACE2 is an integral cell membrane protein that can undergo cleavage and release its catalytically active ectodomain into the environment. A disintegrin and metalloprotease 17 (ADAM17), which is implicated in atherogenesis, was described as the main promoter of ACE2 shedding [58]. Thus, it is possible to conclude that the elevation of ACE2 level in circulation is the consequence of the elevating tissue ACE2 synthesis from mRNA and scaled-ACE2 protein shedding. Taken together, these data highlight the role of elevated levels of circulating ACE2 protein or levels of its activity as atherosclerosis biomarkers. This also stimulates further investigations.

### 5.3. ACE2 as a Therapeutic Target

Human pluripotent stem cells (hPSCs) derived cardiac cells are an important and promising model for COVID-19 investigations. One of the main reasons for such a choice is the fact that ACE2 is not recognized by SARS-CoV-2 in mice. The use of hPSC-derived cardiac cells has already contributed to the finding of fundamental human biology aspects, and also to the numerous human preclinical “trials” for various diseases.

According to COVID-19, this model seems to be extremely beneficial for the viral infections investigations. It even allows testing all kinds of therapies, including the antibody treatment and anti-inflammatory drugs [59].

Despite the need for further investigations, there are already few therapeutic approaches to atherosclerosis targeting ACE2. Thus, captopril and losartan were found to reverse, at least in part, an ACE2 overexpression, which was caused by plasmid-mediated transfection in THP-1 cells and primary monocytes. Such overexpression resulted in a significant reduction of expression of ACE2 mRNA, stimulation of pro-atherogenic phenotype, which is accompanied by an increased gene expression of the cellular adhesion molecules ICAM-1, VCAM-1, and macrophage colony-stimulating factor (MCSF) [60].

Losartan was demonstrated to inhibit the development of atherosclerotic plaques in high-cholesterol fed rabbits and to enhance the production of ACE2 protein within the plaques [61]. Losartan also showed an ability to inhibit both protein expression and activity of ACE2 stimulated by Ang-II. This points at the fact that the ACE2 production can be affected by the generation of Ang-II by ACE, and also that antagonists of AT1R or inhibitors of ACE are able to upregulate ACE2 and stimulate its atheroprotective properties.

Diminazene aceturate (DIZE) is a promising drug acting through the activation of ACE2 [62]. These drug effects were assessed in various animal models indicating its potential for anti-atherosclerotic treatment in humans. Several protective effects were demonstrated for DIZE, among which are decreased lipogenesis and metabolic profile improvement in mice [63], anti-hypertensive effects in renovascular hypertensive rats [64], along with amelioration of the functioning of pulmonary endothelium in Sprague Dawley rats [65]. Ang-II-induced abdominal aortic aneurysms, which are related to atherosclerosis, are defined with the luminal expansion accompanied with extracellular matrix degradation and progressive leukocyte accumulation [66]. The severity of this condition appeared to be decreased by DIZE by Thatcher et al. [67]. Moreover, an enhancement of the stability of atherosclerotic plaques in ApoE-KO mice, as well as reduction of the expression of ICAM-1 and VCAM-1, were described in response to DIZE by Fraga-Silva et al. [68].

However, there are still no clinical evidences of the beneficial effects of these drugs on the COVID-19 clinical course.

It is important to note here, that various drugs and vaccines are already available for the COVID-19 treatment and prevention. Antiviral therapy, immunomodulation, cell and gene therapy, neutralizing antibodies, and other strategies are now used in clinical practice. However, there are still no perfect treatment options.

## 6. Conclusions

Atherosclerosis is a precursor to CVD, which in turn is the most common cause of death in the world. However, at the end of 2019, humanity was faced with a new disease, the prevalence of which quickly assumed the scale of a pandemic.

Even though relatively little time has passed since the onset of COVID-19, we already know a lot about it.

Atherosclerosis and other disorders of the cardiovascular system are risk factors for the development of COVID-19. In turn, COVID-19, can lead to dysfunction of the heart and blood vessels. These consequences include myocarditis, myocardial damage, subsequent heart failure, myocardial infarction, and Takotsubo syndrome.

Although COVID-19, unlike atherosclerosis, is an infectious disease, inflammation and immune response are important components of the pathogenesis of both diseases. RAS is known to play an important role in the maintenance of cardiovascular health, and disturbances in this system lead to serious disorders and unwanted events. A special role in the development of atherosclerosis is assigned to ACE2, which is also a receptor for the Sars-CoV-2 virus, which once again emphasizes the relationship between atherosclerosis and coronavirus disease.

While there is currently no specific treatment for either COVID-19 or atherosclerosis, ACE2 represents an intriguing target for therapeutic development. Among the substances aimed at ACE2 and modulating its expression are losartan, captopril, and DIZE.
